# The Right to Administer Drugs in Dental Practice

**Published:** 1903-10-15

**Authors:** 


### THE RIGHT TO ADMINISTER DRUGS IN DENTAL PRACTICE.

"What legal rights have dentists anyway, outside of plugging, treating or extracting teeth?" is the indignant inquiry of a Pennsylvania correspondent whose legal right to administer anesthetics has been called in question.

To this inquiry but one answer is possible, and that is that the dentist has the same right as the physician to administer systemically or apply locally any drug in the pharmacopeia, provided it is employed in the strict line of his professional work. As with the physician also the right to give drugs carries with it the burden of responsibility for their effect. To this extent both dentists and physicians give drugs at their own risk and are equally subject to penalty for malpractice if what they give proves harmful instead of helpful to the patient.

As the dental law of Pennsylvania expressly requires the the applicant for license shall pass a successful examination before the State Dental Examining Board both in materia medica and anesthesia, and as it is not to be assumed that the State requires a man to know what he is forbidden to practice, the legal right of our correspondent to administer either anesthetics or other drugs for the relief of dental disorders or the prevention of the pain of which they are the occasion is fully fortified by law.

A recent case involving this principle is that of Dr. A. B. Mayhew, of California, which recently came up for trial before Justice Wallace, in San Jose, and the details of which are given in a letter written by Dr. Mayhew's attorney to the editor of the *Pacific Dental Gazette*, from which journal the following excerpts are reproduced:

"Palo Alto is a temperance town and has an ordinance regulating the sale of whisky, etc. Among other provisions, it is by the said ordinance provided, that whisky may be sold by a druggist upon the *prescription* of a *regularly licensed physician.* One George Colpe was arrested and tried for selling whisky to divers persons without a physician's prescription. One Wallace was called as a witness in the aforesaid case and he testified that he went to the office of Dr. Mayhew and told him that he had facial neuralgia; that Dr. Mayhew examined him and confirmed his suspicions; that a doctor then wrote a prescription in which there was mostly whisky; that thereafter the druggist filled the prescription. In other words, our defence to the alleged breach of the ordinance was that a dentist could write a prescription under the circumstances above related.

"We finally won out in the case, and the other side wishing to further embarrass us had Dr. Mayhew arrested and charged with practicing medicine *without* the *license* of a physician, as they thought was provided in the act regulating the practice of medicine passed by the Legislature of 1901. All the evidence they had against Mayhew was the testimony above referred to. The judge did not consider that sufficient, so he dismissed the case before it came to trial on a motion to that effect, that is, the evidence was insufficient to convict.

"If you will read Section 16 of the Act above referred to, especially the part that follows subdivision 4, you will see that a dentist can write a prescription 'when engaged exclusively in the practice of dentistry.' He can prescribe for those diseases with which he has to deal in the course of his business, although he has not a physician's license."

It will thus be seen that in California, as in Pennsylvania, the right of dentists to administer drugs in legitimate dental practice is fully sustained by legal enactment. Even in the absence of specific legislation, however, the principle that the dentist has the right to employ such drugs as are necessary to relieve suffering or produce curative results in dental disorders has, in this country, uniformly been sustained by courts of law.—*Editorial Dental Brief.*

